# Secondary dengue retinitis with associated occlusive retinal vasculitis

**DOI:** 10.1186/s12348-019-0175-x

**Published:** 2019-05-16

**Authors:** Vikram Vinayak Koundanya, Neha Chowdhary, Manisha Agarwal, Prashant Katre

**Affiliations:** Vitreoretina Services, Dr. Shroff’s Charity Eye Hospital, 5027-Kedar Nath Road, Daryaganj, New Delhi, 110002 India

**Keywords:** Secondary dengue retinitis, Occlusive vasculitis, Post-fever retinitis

## Abstract

**Background:**

Dengue is endemic in tropical countries. Secondary dengue infections are generally more dangerous as they lead to an exaggerated response in the patient due to the severe immunological response caused by antibody-dependent enhancement (ADE) leading to severe ocular manifestations like retinitis.

**Results:**

A 42-year-old female was diagnosed as secondary dengue retinitis with associated retinal vasculitis based on her past history, clinical presentation, IgG/IgM ratio, and enzyme-linked immunosorbent assay (ELISA) test for dengue and was successfully treated with oral corticosteroids.

**Conclusion:**

Secondary dengue infection may manifest as retinitis with signs of microvascular occlusions in the retina. A high level of suspicion and IgG/IgM ratio may help in confirming the diagnosis.

## Introduction

Dengue virus belongs to the Flaviviridae family. Secondary dengue infections occur when the patient is affected by a serotype other than the one which caused the primary dengue infection. These infections are generally more dangerous as they lead to an exaggerated immunological response due to antibody-dependent enhancement [1]. The various manifestations of secondary dengue are hemorrhagic fever or dengue shock syndrome and ocular manifestations like sub-conjunctival hemorrhages, retinitis, arteritis with exudation, and vascular sheathing over posterior pole [2, 3]. We report a case of retinitis with associated occlusive retinal vasculitis due to secondary dengue infection which was successfully treated with systemic corticosteroids.

## Case report

**Fig. 1:**
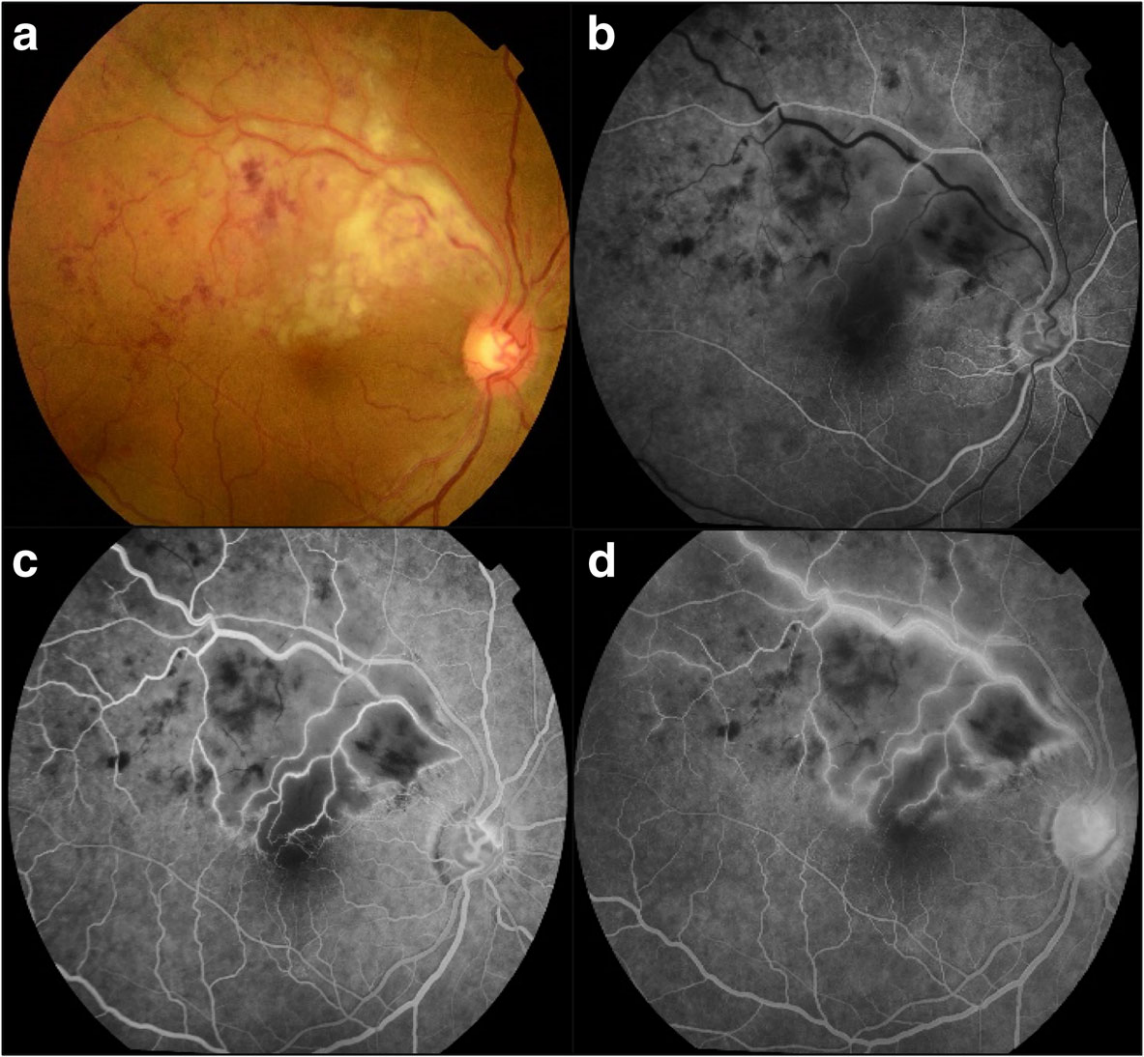
**a** Fundus picture of the right eye showing multiple intra-retinal hemorrhages, venous tortuosity, and dilatation along with retinitis patch (approximately 2 DD) in the superotemporal quadrant. **b** FFA early phase showing hypofluorescence with masking due to intra-retinal hemorrhages and delayed venous filling in the superotemporal quadrant. **c** Mid-phase showing hyperfluorescence along the vessel walls with capillary dropout areas. **d** Late phase showing marked hyperfluorescence along the vessel walls

**Fig. 2:**
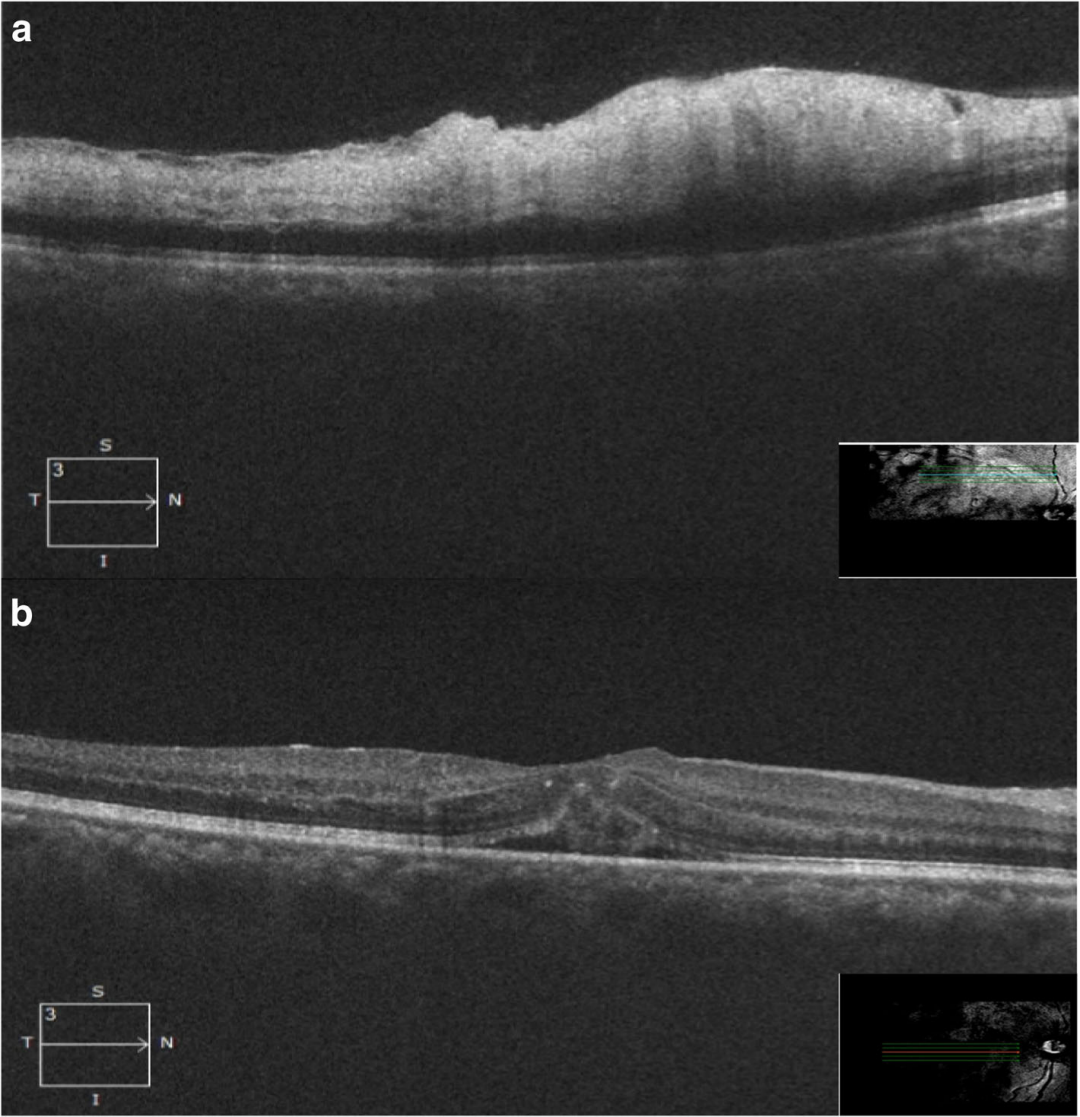
OCT (at presentation): raster scan (**a**) through the lesion showing hyperreflectivity with loss of distinct architecture and increased thickness the inner retinal layers along with sub-ILM fine dot-like deposits. **b** Through fovea showing loss of foveal contour with neurosensory retinal detachment and sub-foveal fluid

A 42-year-old female patient presented with diminution of vision in the right eye for the last 5 days along with myalgia and headache. She had a history of, serology confirmed, dengue fever 7 years back. She also gave a history of two family members suffering from dengue fever for the last 3 weeks. Both were seropositive for dengue. On examination, the best-corrected visual acuity (BCVA) in the right eye was 6/24, N18 and 6/6, N6 in the left eye. Applanation tonometry recorded an intraocular pressure (IOP) of 16 mmHg in both the eyes. Slit lamp examination showed normal anterior segment in both the eyes. There were no cells in the anterior vitreous. Fundus examination of the right eye showed a clear vitreous and dilated and tortuous superotemporal vein with multiple intra-retinal hemorrhages and a patch of retinitis measuring approximately 2-disc diameter along the superotemporal arcade along with a serous detachment of the macula. (Fig. 1a). Left eye fundus was within normal. Fundus fluorescein angiography (FFA) showed normal arm to retina time, areas of blocked fluorescence corresponding to the retinal hemorrhages and early hypofluoresence (Fig. 1b) with late hyperfluorescence (Fig. 1c, d) along the superotemporal arcade and the left eye was within normal. Optical coherence tomography (OCT) of the right eye showed sub-foveal fluid, hyperreflectivity of the inner retinal layers with loss of architecture over the patch of retinitis (Fig. 2). NS-1 antigen test for dengue virus was positive. Serology for dengue IgG was positive while it was negative for Chikungunya, West Nile virus, and yellow fever. Dengue IgG: IgM ratio was 1.8, suggestive of secondary dengue infection.

**Fig. 3:**
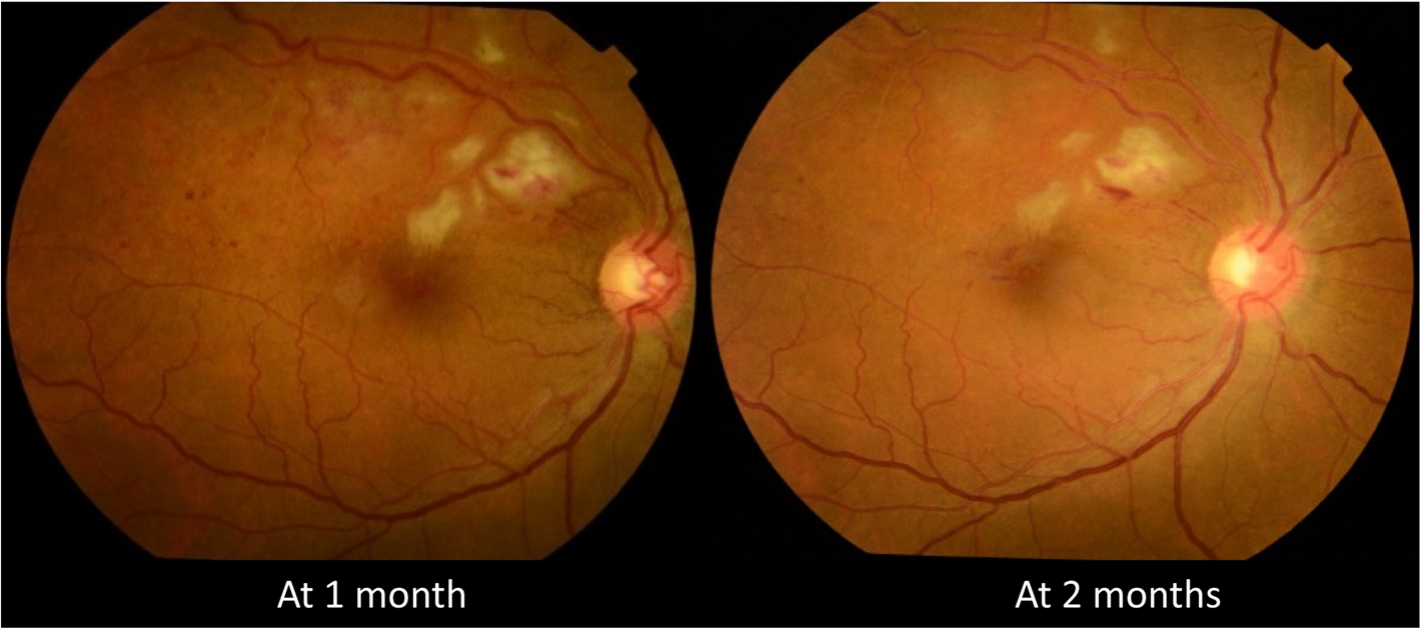
Fundus photograph of the right eye showing the trend in resolution of retinitis post-treatment

**Fig. 4:**
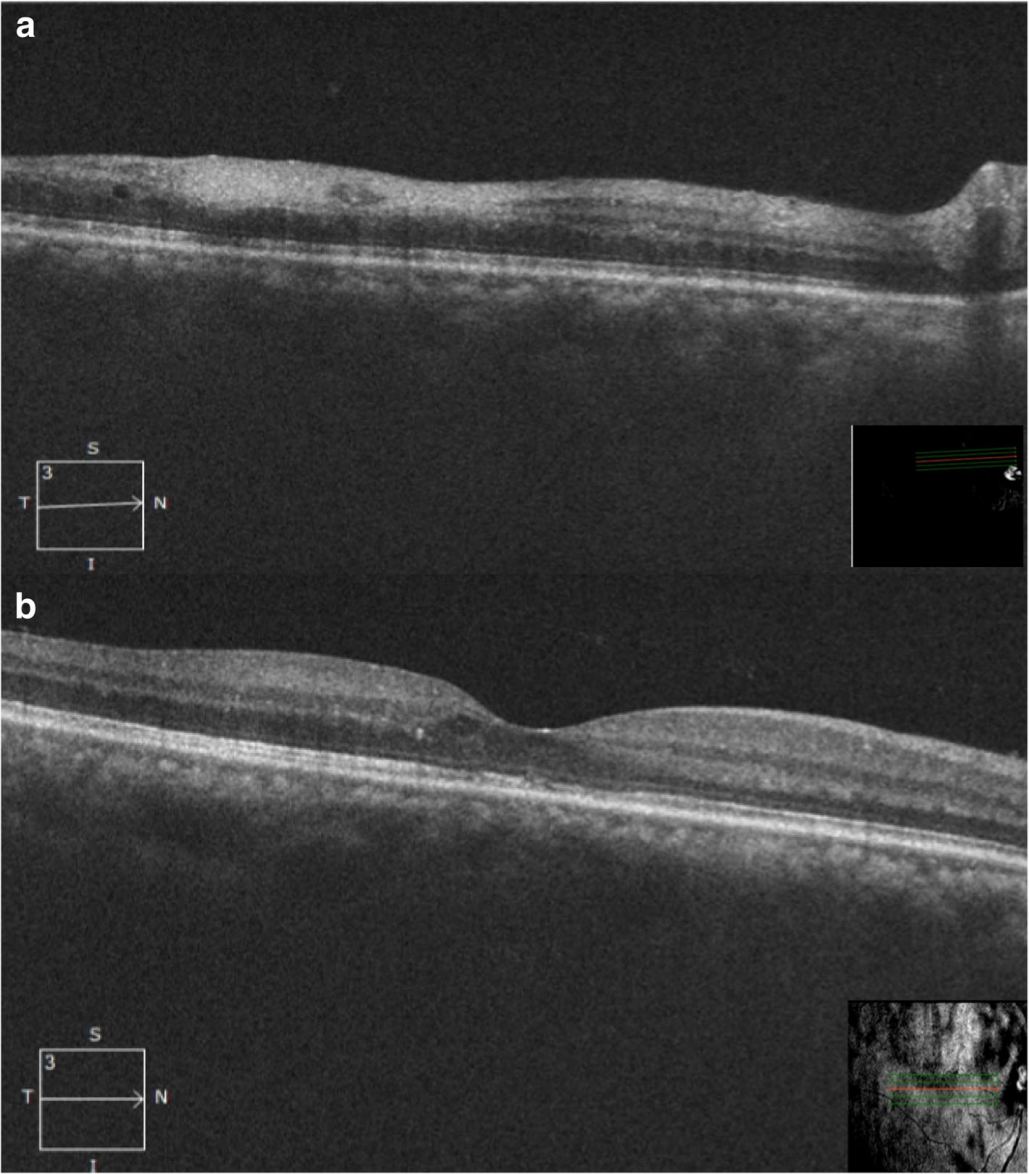
OCT (at 2 months): raster scan (**a**) through the lesion showing resolving edema and decrease in thickness of the inner retinal layers. **b** Through fovea showing normal foveal contour with residual discrete cystoid spaces

A clinical diagnosis of dengue retinitis was made, and the patient was started on oral corticosteroids (1 mg/kg). At 4 weeks follow-up, the BCVA in the right eye was 6/6,N6. Fundus examination of the right eye showed resolving retinal hemorrhages and retinitis patch (Fig. 3b). The patient was advised to continue oral steroids in tapering dose and was asked to review after 1 month. Oral steroids were given for a total of 6 weeks in tapering dose. Follow-up at 2 months showed further improvement in retinitis (Fig. 3c). OCT of the right eye showed a decrease in the thickness of the inner retinal layers and resolving edema (Fig. 4).

## Discussion

Dengue virus is a member of the *Flaviviridae* family, genus *Flavivirus.* Four distinct serotypes (DEN-1, DEN-2, DEN-3, and DEN-4) of dengue virus have been identified. Infection with any of these viruses may be asymptomatic or cause a self-limiting febrile illness known as dengue fever. Although rare, dengue retinitis has been reported after acute dengue fever [3]. Typically, it presents 2–3 weeks after an attack of acute dengue fever. Other reported complications associated with dengue are retinochoroiditis [4], choroiditis [5], optic neuritis [6], central retinal artery occlusion [7], and panophthalmitis [8].

Infection with one serotype of dengue virus imparts a lifelong immunity against that particular serotype. Infection with other serotype or multiple infections with different serotypes of dengue virus in a person who was infected with another serotype in the past is called secondary dengue [9].

Pathogenesis in dengue is linked to the host immune response, which is triggered by infection with the virus. Primary infection is usually benign. Severe infection can be caused by secondary infection with a different serotype or multiple infections with different serotypes. The IgG capture ELISA could be used to distinguish between primary and secondary infection, with 100% of primary infections and 96% of secondary infections being correctly classified. Primary and secondary dengue infections may be classified by determining the ratio of units of dengue IgM to IgG antibody [9]. IgG/IgM ratio of > 1.10 is useful diagnosing secondary dengue [10].

Our patient had no manifestation of active dengue fever but had a past history of seropositive dengue fever 7 years back. This would have apparently imparted lifelong immunity in her for the serotype of dengue virus which infected her 7 years back. However, the history of two of her family members suffering from dengue fever raised the suspicion of secondary dengue infection in her. On investigations, her IgG/IgM ratio was 1.8 which confirmed the diagnosis of secondary dengue in her.

Numerous reports have identified a second heterologous dengue virus (DENV) infection as a principal risk factor for severe dengue disease (dengue hemorrhagic fever/dengue shock syndrome, DHF/DSS). Dengue cross-reactive antibodies raised following a first dengue infection combined with a second infecting virus to form infectious immune complexes that enter Fc-receptor-bearing cells [10]. This results in an increased number of infected cells and high level of cytokines leading to severe complications.

To the best of our knowledge, this is the first case of secondary dengue retinitis reported.

## Conclusion

We report this rare case to highlight the fact that absence of fever in a patient may be misleading to the treating doctor and the patient may be suffering from secondary dengue infection which may manifest as retinitis with signs of microvascular occlusions in the retina. A high level of suspicion and IgG/IgM ratio may help in confirming the diagnosis. Early diagnosis and management may help in complete recovery of vision in such eyes.

## Abbreviations

ADE: Antibody-dependent enhancement
BCVA: Best-corrected visual acuity
DENV: Dengue virus
DHF: Dengue hemorrhagic fever
DSS: Dengue shock syndrome
ELISA: Enzyme-linked immunosorbent assay
FFA: Fundus fluorescein angiography
IOP: Intraocular pressure
NS1: Non-structural protein 1
OCT: Optical coherence tomography
RPE: Retinal pigment epithelium
